# Evaluation of diaphragm thickness to predict intubation requirement and mortality in critical COVID-19 patients

**DOI:** 10.15537/smj.2022.43.10.20220469

**Published:** 2022-10

**Authors:** Hayriye C. Dal, Betul A. Dolek, Muhammet A. Beyoğlu, Dilek Acar, Melih G. Gozukara, Sema Turan

**Affiliations:** *From the Department of Intensive Care Unit (Dal, Turan); from the Department of Radiology (Dolek, Acar); from the Department of General Thoracic Surgery (Beyoğlu), University of Health Sciences, Ankara City Hospital, and from the Department of Public Health (Gozukara), Ankara Sincan Health Directorate, Ankara, Turkey.*

**Keywords:** COVID-19, diaphragm, computed tomography, mortality, mechanical ventilation

## Abstract

**Objectives:**

To investigate the value of measuring the diaphragm thickness (DT) on thorax computed tomography (CT) at intensive care unit (ICU) admission for predicting intubation requirement and mortality among COVID-19 patients.

**Methods:**

This study was carried out in Ankara City Hospital, Ankara, Turkey, from September 2020 to January 2021, with 94 critical COVID-19 patients. The patients’ demographic characteristics, laboratory parameters, DT measurements, mechanical ventilation (MV) requirements, and mortality statuses were retrospectively screened. The relationships between DT on initial CT, MV requirement, and mortality were investigated.

**Results:**

Diaphragm thickness was lower in patients who required intubation after ICU admission than in non-intubated patients (*p*=0.006); it was also lower in non-survivors (*p*=0.009). The threshold values for MV need was 3.35 mm (*p*=0.004) and 3.275 mm for mortality (*p*=0.006), according to the receiver operating characteristic analysis used to assess the predictive potential of DT. The non-survivor group had a greater neutrophil-to-lymphocyte ratio (*p*=0.026). Absolute neutrophil count (*p*=0.017), neutrophil-to-lymphocyte ratio (*p*=0.010), and interleukin-6 levels (*p*=0.027) were higher among patients requiring MV than among non-intubated patients.

**Conclusion:**

Mortality and MV requirements can be predicted from DT measurements. Diaphragm thickness can facilitate the identification of high-risk patients on CT evaluation at ICU admission.

**I**mpacting the entire world, COVID-19 was declared a pandemic in early 2020 and is still present worldwide. The disease progresses with mild flu symptoms or is even asymptomatic in some cases, but it can cause severe respiratory failure in others.^1^ Around the world, more than 470 million individuals have been infected with COVID-19 to date, and 6 million have died as a result of the disease.^2^ Most intensive care unit (ICU) beds worldwide have been allocated for COVID-19 because severe cases necessitate an ICU stay. The course of COVID-19 is primarily associated with respiratory system involvement, and patients admitted to the ICU usually develop respiratory failure. Non-invasive oxygen support can be adequate in some cases, whereas invasive mechanical ventilation (MV) is necessary for the critical ones.^3^

Since the beginning of the pandemic, numerous studies have investigated the use of laboratory parameters and imaging methods for the early diagnosis of COVID-19, defining general disease characteristics, determining the severity of cases, and estimating hospitalization and mortality; various scoring systems have also been proposed.^4-6^ Studies on ICU patients, however, have mostly been limited to the evaluation of laboratory parameters.

Approximately 65-80% of vital capacity is produced by the diaphragm, the major muscle of ventilation. The muscle’s dysfunction causes alveolar hypoventilation, which, in critical cases, can progress to respiratory failure requiring MV. In these cases, increased respiratory demands or deteriorating diaphragm function leads to inappropriate activation of the rib cage and abdominal expiratory muscles, leading to breathing difficulty.^7^ Various methods, such as direct radiography, fluoroscopy, thorax computed tomography (CT), and ultrasonography (USG), are used for imaging the diaphragm. Among these, CT is known to be superior in anatomical evaluation of the muscle and in detecting relevant pulmonary diseases.^8^ The previous literature includes studies that evaluated diaphragm thickness (DT) among COVID-19 patients with USG, but to our knowledge, there is limited information on DT evaluation with CT.

A study by Corradi et al^9^ evaluating DT with USG in COVID-19 patients stated that low DT poses a risk in terms of intubation and adverse outcomes. However, the study involved non-critical patients rather than ICU patients. Also the radiologist’s close contact with the patient during USG imaging poses a risk of COVID-19 transmission. Therefore, it can be more practical to evaluate DT on thorax CT, which is routinely carried out in critical COVID-19 cases.

Thorax CT is valuable for diagnosing COVID-19, evaluating pulmonary involvement, and predicting prognosis.^10^ It is routinely carried out in the initial evaluation of severe cases. The present study investigates the value of measuring DT on thorax CT at ICU admission for predicting MV requirement and adverse outcomes, such as mortality, among COVID-19 patients.

## Methods

In a tertiary-level hospital in Ankara, Turkey, a retrospective, observational study at a single center was carried out. The Clinical Research Ethics Committee of the University of Health Sciences, Ankara City Hospital, Ankara, Turkey, approved the study protocol (approval no. 2021/E2-21-390). After approval was obtained, all data were accessed from electronic medical records and patient files. The Declaration of Helsinki’s principles were followed.

The study population included patients over 18 years of age, positive for COVID-19 (SARS-CoV-2) reverse transcriptase polymerase chain reaction (PCR), who were hospitalized in the ICU between September 2020 and January 2021. Exclusion criteria consisted of an ICU stay less than 24 hours, invasive or non-invasive MV before ICU admission, absence of a thorax CT, and the presence of chronic respiratory disorders.

We created a database of every piece of information regarding the patient, including their demographics, comorbidities, date of ICU admission, Acute Physiology and Chronic Health Evaluation II (APACHE-II) and Glasgow Coma Scale (GCS) scores, laboratory results, DT measures, need for invasive MV, MV time, ICU mortality, and length of stay in the ICU. An English-language electronic literature search of MEDLINE was carried out to identify prior related studies.

The following scan settings were used: 100 kV, 110 mAs, body filter, 1.25 mm slice thickness, 512x512 reconstruction matrix, and spiral pitch factor 1.375:1 on a 128 slice CT (GE Revolution EVO, Milwaukee, WI, USA) during inspiration without intravenous contrast.

**Figure 1:**
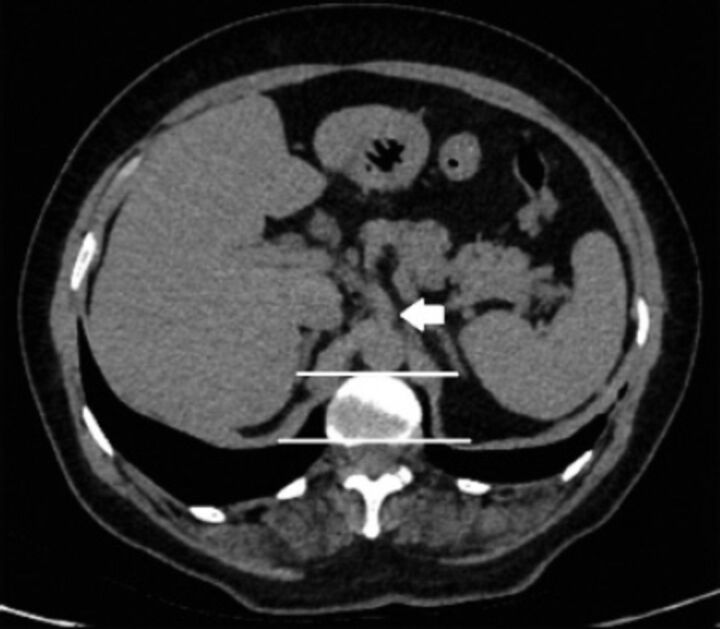
- Points of diaphragm thickness measurements. Axial computed tomography scan at the celiac artery origin level (white arrow). Anterior and middle diaphragm thickness measurement points (white lines).

All CT images were evaluated on the PACS workstation (Advantage Windows Workstation, version 4.7, GE Healthcare). Two radiologists took the measures 3 times on each side of the diaphragm, and the magnification was freely modifiable. For each set of measurements, the mean value was determined. During CT scans, the radiologists were blinded to patient information such as clinical characteristics and outcomes. The mediastinal window was carried out to assess CT scans (window center, 90 HU; window width, 350 HU). Axial images were used to measure the diaphragmatic crura at the celiac artery’s origin and record all measures for each crus at the anterior and central sides of the vertebral body (Figure 1).

### Statistical analysis

The Statistical Package for the Social Sciences, version 23.0 (IBM Corp., Armonk, NY, USA) was carried out. The variables were assessed visually (using probability plots and histogram), and the Shapiro-Wilk test showed that the distribution was non-parametric. Demographic data are expressed as number and precentage (%) and median (minimum-maximum). The Mann-Whitney-U test was carried out to compare the quantitative values of 2 independent groups due to the non-parametric distribution. The receiver operating characteristic (ROC) curve for the MV requirement and mortality was used to calculate the DT cut-off values. The 95% confidence interval (CI) and area under the ROC curve (AUC) were computed. Statistical significance was defined as *p*<0.05.

## Results

The study included 94 COVID-19 patients who stayed in the ICU and met the study criteria. The median age was 69 (27-90) years. The patients’ demographic details and comorbidities are shown in Table 1. The median GCS score was 15, and the APACHE-II score was 17 (3-15). The overall mortality rate was 53.2%, and the median length of stay in the ICU was 9 (2-58) days.

**Table 1:** - Clinical and demographic characteristics of the patients (N=94).

Variables	n (%)
***Age***	
0-64 65-74 75-84 85 and above	37 (39.4) 27 (28.7) 20 (21.3) 10 (10.6)
***Gender***	
Male Female	36 (38.3) 58 (61.7)
***Comorbidities***	
None Hypertension Type 2 diabetes mellitus Cardiovascular disease Malignity Chronic kidney failure Cerebrovascular disease	14 (14.9) 50 (53.2) 27 (28.7) 29 (30.9) 7 (7.4) 4 (4.3) 8 (8.5)
***Respiratory support of the patients***	
Nasal cannula Simple mask Non-rebreathing mask High flow nasal oxygen NIMV MV	7 (7.4) 5 (5.3) 11 (11.7) 7 (7.4) 8 (8.6) 56 (59.6)

Values are presented as a number and precentage (%). NIMV: noninvasive mechanical ventilation, MV: mechanical ventilation

Laboratory parameters and other demographic and clinical data at ICU admission were compared between the non-survivors and survivors. In the non-survivor group, the APACHE-II score was higher, the MV time was longer, and the GCS score was lower (*p*<0.001). The non-survivor group’s neutrophil-to-lymphocyte ratio (NLR) at admission was higher (*p*<0.026) than that of the survivor group. Diaphragm thickness was lower in the non-survivor group (2.95 mm [1.00-6.40]) than in the survivor group (3.41 mm [1.75-5.78]) (*p*=0.009; Table 2). A total of 56 (59.6%) patients required intubation during their ICU stay and underwent MV. The median MV time was 2.5 (0-58) days. Table 1 shows the non-invasive oxygen support modalities applied in other patients. Diaphragm thickness was lower among patients requiring invasive MV during their ICU stay (3.03 mm [1.00-6.40]) than among non-intubated patients (3.46 mm [1.75-5.78]; *p*=0.006). Regarding laboratory parameters, the patients requiring MV also had higher levels of NLR (*p*=0.010), absolute neutrophil count (ANC; *p*=0.017), and interleukin-6 (IL-6; *p*=0.027; Table 3).

**Table 2:** - Clinical characteristics and laboratory parameters of survivor and nonsurvivor patients (N=94).

Variables	ICU survivors (n=44)	ICU nonsurvivors (n=50)	*P*-values*
Age	32.5 (27-87)	72 (41-90)	<0.001
***Gender, n (%)***			
Female Male	11 (30.6) 33 (56.9)	25 (69.4) 25 (43.1)	0.013
APACHE-II score	10 (2-18)	26.5 (11-50)	<0.001
GCS	15 (3-15)	13 (3-15)	<0.001
MV duration days	0 (0-36)	5 (1-58)	<0.001
LOS ICU (days)	6.5 (2-43)	10.5 (2-58)	0.062
Hemoglobin (gr/dL)	12.7 (7.5-16.1)	12.65 (8-15.7)	0.655
WBC (x10^9^/L)	8.92 (2.83-24.67)	9.05 (0.59-39.90)	0.218
Lymphocyte (x10^9^/L)	0.735 (0.20-3.91)	0.64 (0.80-2.59)	0.102
Neutrophile (x10^9^/L)	7.18 (2.17-22.98)	8.11 (0.44-36.0)	0.056
NLR	8.6 (0.06-31.0)	12.41 (0.95-90.44)	0.026
Platelet (x10^9^/L)	288 (81-662)	233.5 (38-464)	0.234
D-Dimer (mg/L)	2.02 (0.30-35.2)	2.8 (0.4-35.2)	0.342
CRP (g/L)	0.092 (0.005-0.293)	0.139 (0.008-0.740)	0.099
Procalcitonin (µg/L)	0.165 (0.03-27.70)	0.180 (0.03-32.40)	0.285
Ferritin (µg/L)	733.0 (28-19000)	741.0 (77-7934)	0.880
IL-6 (pg/ml)	46.1 (4.7-235.0)	72.00 (4.99-8490.0)	0.022
AST (U/L)	41.5 (4-360)	48.0 (6-630)	0.195
ALT (U/L)	35.0 (6-89)	36.5 (8-490)	0.773
Creatinine (mg/dL)	0.88 (0.40-10.72)	0.99 (0.46-43.0)	0.544
***Diaphragm thickness (mm)***			
DT anterior right DT anterior left DT middle right DT middle left Total median DT	72 (41-90) 3.75 (1.6-7.7) 2.95 (1.3-5.1) 2.6 (0.8-4.9) 3.41 (1.75-5.78)	62.5 (27-87) 2.9 (0.9-6.2) 2.3 (1.2-6.5) 2.25 (0.7-6.1) 2.95 (1.00-6.40)	0.009 0.014 0.038 0.054 0.009

* Variables with non-normal distribution are presented as median (minimum-maximum) and Mann-Whitney-U test was used. APACHE-II: acute physiology and chronic health evaluation-II, GCS: Glasgow coma scale, MV: mechanical ventilation, LOS: length of stay, ICU: intensive care unit, WBC: white blood cell, NLR: neutrophil to lymphocyte ratio, CRP: C-reactive protein, IL-6: interleukin-6, AST: aspartate aminotransferase, ALT: alanine aminotransferase, DT: diaphragm thickness

**Figure 2:**
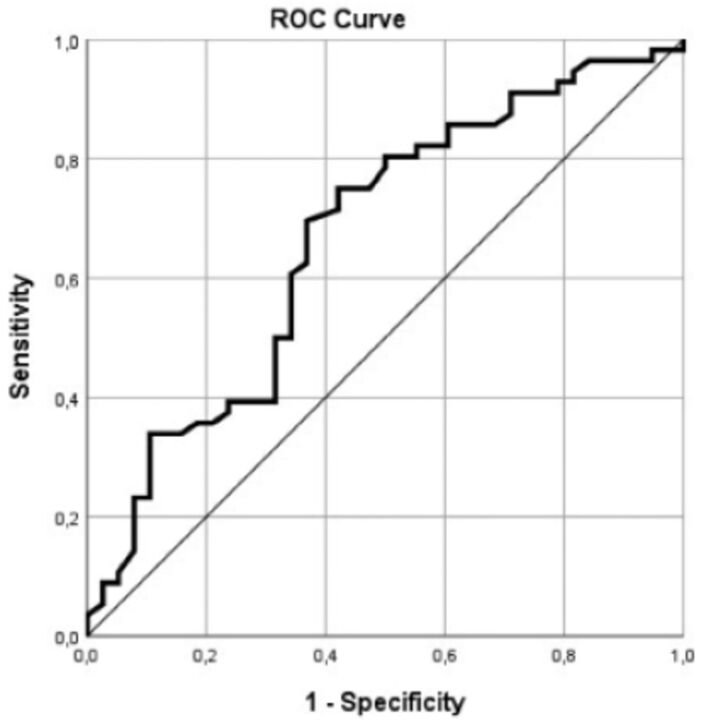
- Receiver operating characteristic (ROC) curve of total diaphragm thickness (DT) to predict the need for invasive mechanical ventilation. Receiver operating characteristic analysis to evaluate the predictive power of DT revealed a cut-off value of 3.35 mm (AUC: 0.666, 95% CI: [0.561-0.760]; *p*=0.004) for invasive mechanical ventilation requirement. AUC: area under the curve, CI: confidence interval

**Figure 3:**
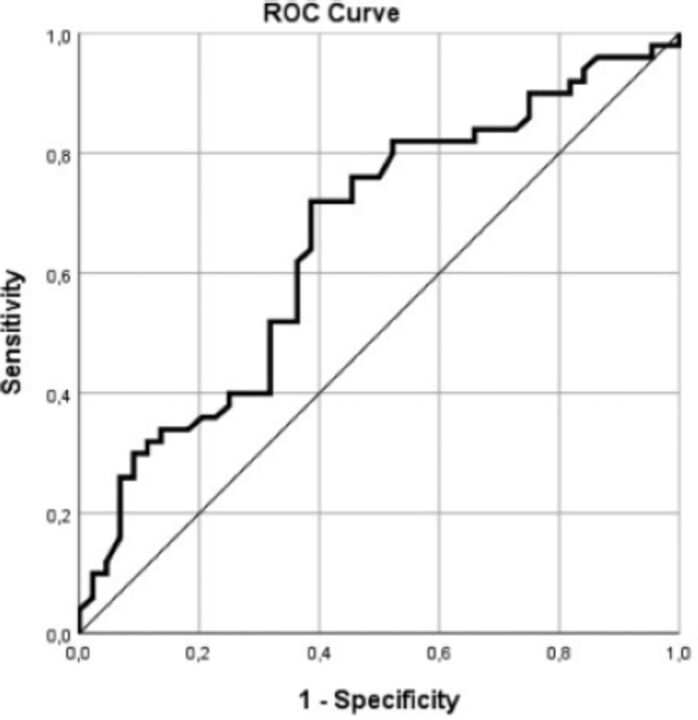
- Receiver operating characteristic (ROC) curve of total diaphragm thickness (DT) to predict the mortality. Receiver operating characteristic analysis to evaluate the predictive power of DT revealed a cut-off value of 3.275 mm (AUC: 0.656, 95% CI: [0.551-0.751]; *p*=0.006) for mortality. AUC: area under the curve, CI: confidence interval

Our ROC analysis to evaluate the predictive power of DT revealed a cut-off value of 3.35 mm (AUC: 0.666, 95% CI: [0.561-0.760]; *p*=0.004) with a sensitivity of 75.00 and a specificity of 57.89 for invasive MV requirement (Figure 2), and 3.275 mm (AUC: 0.656, 95% CI: [0.551-0.751]; *p*=0.006) with a sensitivity of 72.00 and a specificity of 61.36 for mortality (Figure 3).

**Table 3:** - Clinical characteristics and laboratory parameters of mechanical ventilation and non-mechanical ventilation patients (N=94).

Variables	Non-MV (n=38)	MV (n=56)	*P*-values*
Age	62.5 (27-87)	72 (34-90)	0.002
***Gender, n(%)***			
Female Male	10 (27.8) 28 (48.3)	26 (72.2) 30 (51.7)	0.049
APACHE-II score	8.5 (2-18)	25 (7-50)	<0.001
GCS	15 (8-15)	13 (3-15)	<0.001
LOS ICU (days)	6 (2-25)	11.5 (2-58)	<0.001
Hemoglobin (gr/dL)	12.95 (7.5-16.1)	12.55 (8.0-15.7)	0.322
WBC (x10^9^/L)	8.54 (2.83-18.94)	9.48 (0.58-39.90)	0.059
Lymphocyte (x10^9^/L)	0.72 (0.21-3.91)	0.66 (0.08-2.59)	0.162
Neutrophile (x10^9^/L)	6.82 (2.17-17.55)	8.11 (0.44-36.0)	0.017
NLR	8.55 (1.91-31.0)	12.55 (0.06-90.44)	0.010
Platelet (x10^9^/L)	273.5 (85-662)	240.5 (38-598)	0.535
D-Dimer (mg/L)	1.81 (0.30-35.2)	2.8 (0.40-35.2)	0.126
CRP (g/L)	0.081 (0.005-0.293)	0.134 (0.008-0.740)	0.058
Procalcitonin (µg/L)	0.155 (0.03-27.70)	0.180 (0.03-32.40)	0.174
Ferritin (µg/L)	566.5 (28-19000.0)	788.5 (77-7934)	0.523
IL-6 (pg/ml)	43.15 (4.7-235.0)	66.9 (4.99-8490.0)	0.027
AST (U/L)	41.5 (4-360)	48.0 (6-630)	0.189
ALT (U/L)	35 (6-89)	36.5 (8-490)	0.689
Creatinine (mg/dL)	0.90 (0.61-10.72)	0.95 (0.40-43.0)	0.644
***Diaphragm thickness (mm)***			
DT anterior right DT anterior left DT middle right DT middle left Total median DT	4.35 (2.1-6.8) 3.8 (1.6-7.7) 3.25 (1.3-5.1) 2.6 (0.8-4.9) 3.46 (1.75-5.78)	3.6 (1.2-7.1) 3.6 (0.9-6.2) 2.4 (1.2-6.5) 2.25 (0.7-6.1) 3.03 (1.00-6.40)	0.009 0.014 0.024 0.034 0.006

* Variables with non-normal distribution are presented as median (minimum-maximum) and Mann-Whitney-U test was used. MV: mechanical ventilation, APACHE-II: acute physiology and chronic health evaluation-II, GCS: Glasgow coma scale, MV: mechanical ventilation, LOS: length of stay, ICU: intensive care unit, WBC: white blood cell, NLR: neutrophil to lymphocyte ratio, CRP: C-reactive protein, IL-6: interleukin-6, AST: aspartate aminotransferase, ALT: alanine aminotransferase, DT: diaphragm thickness

## Discussion

Despite the decrease in mortality rates thanks to vaccination programs, the COVID-19 pandemic has not ended yet, and its impact continues. Therefore, identifying risk factors during ICU admission is crucial to prevent adverse outcomes in critical cases. In this study, we evaluated DT on thorax CTs obtained at ICU admission in critical COVID-19 cases and observed that low DT was associated with high MV requirement and mortality. Thorax CT is commonly used to evaluate lung parenchymal involvement in COVID-19 before ICU admission, but DT is not routinely examined in these CT scans. Evaluating the diaphragm, which is the major muscle of respiration, can prove valuable in predicting poor prognosis at an early phase.

Diaphragm thickness can decrease due to various reasons, and invasive MV requirement is higher among patients with low DT due to insufficient respiratory compensation.^9^ Although low DT is not a direct diaphragmatic pathology, increased respiratory workload could disrupt ventilation. A study evaluating respiratory muscles and the diaphragm reported a DT of 4.1-4.4 mm in healthy adults.^11^ Our analysis of the ability to predict DT revealed a cut-off value of 3.35 mm (95% CI, sensitivity 75.00, specificity 57.89) for invasive MV requirement and 3.275 mm (95% CI, sensitivity 72.00, specificity 61.36) for mortality. Therefore, assessing DT at ICU admission can enable prompt detection of patients who might benefit from close monitoring and early intervention. Furthermore, actions to counteract sarcopenia, application of respiratory physiotherapy, and optimization of nutrition to prevent diaphragm dysfunction can prevent adverse outcomes. In a study evaluating the value of DT to predict reintubation in extubated ICU cases, Ni et al^12^ reported that a value of ≥1.55 mm decrease in DT was statistically significant for increased reintubation and mortality.

Diaphragm thickness can be examined using various imaging methods. Thorax CT and USG are the primary methods used to observe the diaphragm. Recently, USG has been widely carried out in ICU patients thanks to its bedside application and ease of use. Various non-COVID-19 studies have emphasized the efficacy of USG in evaluating diaphragmatic weakness associated with critical respiratory disease.^13,14^ However, USG evaluation is dependent on the physician and can considerably vary, and, unlike CT, it does not allow for visualization of the entire diaphragm.^15^ In a prospective study evaluating diaphragmatic excursion with USG carried out within the first 12 hours of ICU hospitalization in critically ill COVID-19 patients, it was reported that low diaphragmatic excursion was significant in predicting the need for intubation and mortality. However, because a single doctor in this study carried out the diaphragm evaluation, it was stated that further studies are needed to determine a diaphragmatic excursion cut-off value for mortality and predicting the intubation requirement.^16^ Furthermore, the radiologist’s close contact with the patient during USG imaging poses a risk of COVID-19 transmission. Therefore, it can be more practical to evaluate DT on thorax CT, which is routinely obtained in critical COVID-19 cases.

There is no consensus on where to measure DT on a CT scan. In a relevant study by Ufuk et al,^17^ CT scans were evaluated by 4 radiologists, and the diaphragm crura were measured at 5 different points. According to the best intra-observer and inter-observer agreement, the authors recommended measuring celiac artery outlet level for maximum DT and the upper level of the L1 vertebral body for the anterior DT.^17^ Another CT study suggested using the method of drawing the line at the celiac artery level and the anterior border of the vertebral canal in axial images and measuring from the point on axial and coronal images where the line intersects with the other lines coming from the both sides of the diaphragm on the transverse plan.^18^ In light of the literature, we measured diaphragmatic crura at the origin of the celiac artery and recorded measurements for each crus at the anterior and middle aspect of the vertebral body on axial images.

Although there are no direct indications for diaphragmatic dysfunction in COVID-19, studies have shown that increased oxidative stress and elevated cytokine levels in critical non-COVID-19 patients can cause diaphragm dysfunction in the event of systemic inflammation.^19^ Coronavirus disease-19 is known to cause hyperinflammation and cytokine storm; thus, pro-inflammatory cytokines could cause sarcolemma damage and diaphragm atrophy by proteolysis.^20^ This is one of the main reasons we investigated diaphragm dysfunction in patients with COVID-19 in the present study.

Neutrophil-to-lymphocyte ratio is a laboratory parameter that becomes elevated in systemic inflammation and has been associated with infection and sepsis-related mortality.^21,22^ As it is a part of routine hemogram tests, this parameter can be easily evaluated. There are many studies underlining the importance of NLR, ANC, and IL-6 levels in determining disease severity and estimating prognosis in COVID-19. Increased NLR and IL-6 levels, especially in the hyperinflammatory phase, are reported to be indicators of mortality.^23-25^ In our study, high NLR was found to be significant for mortality, and high NLR, ANC, and IL-6 for invasive MV requirement. Evaluating NLR levels together with DT can provide accuracy in predicting mortality in COVID-19 cases.

To the best of our knowledge, there is limited information in the literature on evaluating DT with thorax CT in critical COVID-19. A pilot study evaluating DT with USG in COVID-19 patients stated that a DT value of <2.2 mm occurring in the first 36 hours of hospitalization poses a risk in terms of intubation and adverse outcomes. Furthermore, low DT and lymphopenia were indicated as independent variables to estimate mortality. However, the study involved non-critical patients rather than ICU patients.^9^ The value of our investigation lies in the fact that our study population consisted of ICU patients, that is, severe cases for whom the risk factors for MV requirement and adverse outcomes are directly related.

Published studies on DT evaluation by CT in the ICU have generally focused on intubated patients. For instance, Lee et al^18^ examined thorax CTs obtained 18.4±14.9 days apart during the ICU stay of patients receiving MV and measured a mean left side DT of 3.8±0.6 and right side DT of 3.9±0.8 mm, in the first scans, and 3.4±0.6 and 3.5±0.9 mm, in the second scans (*p*<0.05). The authors concluded that MV causes a reduction of DT in critical patients. Various studies, including studies on animal models, have revealed that pressures during MV cause diaphragm fiber injury, resulting in decreased DT.^26,27^ In our study, we excluded patients who were intubated in the emergency clinic or another service before ICU admission to rule out this known effect of MV on DT. Patients with chronic respiratory diseases such as COPD and asthma were also excluded because the focus of our study was COVID-19.

Evaluating DT to detect high-risk cases before ICU admission can allow for controlled use of the practices linked with diaphragm dysfunction, such as sedation and steroid usage, and thus prevent muscle weakness leading to respiratory failure.^28,29^ Furthermore, taking measures such as optimization of nutrition and respiratory physiotherapy in cases at risk of diaphragmatic atrophy during ICU stay can reduce invasive MV requirement and adverse outcomes.

### Study limitations

The retrospective methodology, limited patient population, and that it is a single-center design. Despite the study’s shortcomings, we believe it will shed light on future studies on the development of a cut-off for DT, especially in predicting intubation, on this subject, which has limited information in the literature. With the studies to be carried out on this subject, it could be a guide for the use of DT evaluation on CT obtained during hospitalization in critical COVID-19 cases during the pandemic process, which is still ongoing, in clinical practice.

In conclusion, measuring DT on thorax CT at ICU admission can be predictive of MV requirement and mortality among COVID-19 cases. It should be considered that a thin DT might be associated with poor outcomes in patients. Evaluation of DT could help identify high-risk patients who might benefit from close monitoring.
